# The causal relationship between genetically predicted blood metabolites and idiopathic pulmonary fibrosis: A bidirectional two-sample Mendelian randomization study

**DOI:** 10.1371/journal.pone.0300423

**Published:** 2024-04-16

**Authors:** Tingyu Pan, Le Bai, Dongwei Zhu, Yun Wei, Qi Zhao, Fanchao Feng, Zhichao Wang, Yong Xu, Xianmei Zhou

**Affiliations:** 1 Department of Pulmonary and Critical Care Medicine, Jiangsu Province Hospital of Chinese Medicine, Affiliated Hospital of Nanjing University of Chinese Medicine, Nanjing, Jiangsu, China; 2 School of Chinese Medicine, School of Integrated Chinese and Western Medicine, Nanjing University of Chinese Medicine, Nanjing, Jiangsu, China; Xi’an Jiaotong University Medical College First Affiliated Hospital Department of Medical Oncology, CHINA

## Abstract

**Background:**

Numerous metabolomic studies have confirmed the pivotal role of metabolic abnormalities in the development of idiopathic pulmonary fibrosis (IPF). Nevertheless, there is a lack of evidence on the causal relationship between circulating metabolites and the risk of IPF.

**Methods:**

The potential causality between 486 blood metabolites and IPF was determined through a bidirectional two-sample Mendelian randomization (TSMR) analysis. A genome-wide association study (GWAS) involving 7,824 participants was performed to analyze metabolite data, and a GWAS meta-analysis involving 6,257 IPF cases and 947,616 control European subjects was conducted to analyze IPF data. The TSMR analysis was performed primarily with the inverse variance weighted model, supplemented by weighted mode, MR-Egger regression, and weighted median estimators. A battery of sensitivity analyses was performed, including horizontal pleiotropy assessment, heterogeneity test, Steiger test, and leave-one-out analysis. Furthermore, replication analysis and meta-analysis were conducted with another GWAS dataset of IPF containing 4,125 IPF cases and 20,464 control subjects. Mediation analyses were used to identify the mediating role of confounders in the effect of metabolites on IPF.

**Results:**

There were four metabolites associated with the elevated risk of IPF, namely glucose (odds ratio [OR] = 2.49, 95% confidence interval [95%CI] = 1.13–5.49, *P* = 0.024), urea (OR = 6.24, 95% CI = 1.77–22.02, *P* = 0.004), guanosine (OR = 1.57, 95%CI = 1.07–2.30, *P* = 0.021), and ADpSGEGDFXAEGGGVR (OR = 1.70, 95%CI = 1.00–2.88, *P* = 0.0496). Of note, the effect of guanosine on IPF was found to be mediated by gastroesophageal reflux disease. Reverse Mendelian randomization analysis displayed that IPF might slightly elevate guanosine levels in the blood.

**Conclusion:**

Conclusively, hyperglycemia may confer a promoting effect on IPF, highlighting that attention should be paid to the relationship between diabetes and IPF, not solely to the diagnosis of diabetes. Additionally, urea, guanosine, and ADpSGEGDFXAEGGGVR also facilitate the development of IPF. This study may provide a reference for analyzing the potential mechanism of IPF and carry implications for the prevention and treatment of IPF.

## Introduction

Idiopathic pulmonary fibrosis (IPF) is a chronic progressive disorder characterized by fibrosing interstitial pneumonia, dyspnea, lung dysfunction, and compromised quality of life [1], which predominantly affects the elderly. Despite the existence of pharmacological interventions such as pirfenidone and nintedanib, IPF is still a fatal disorder with a median survival of only 3.8 years [2]. The risk of IPF can be elevated by several factors, such as chronic viral infections, cigarette smoking, exposure to diverse kinds of dust and fumes, and genetic susceptibility [3]. The comprehension of IPF pathogenesis has steadily evolved from an inflammation-driven mechanism [4] to speculation highlighting aberrant alveolar epithelial cell activation [5]. Nevertheless, the definite etiology of IPF is still ambiguous.

Metabolomics is an emergent and burgeoning high-throughput technique for the identification of small molecules within biological samples, which creates a fresh avenue for discovering disease mechanisms [6]. Metabolites are the downstream product of preceding genes and proteins and reflect the current state of individuals. Of note, accumulating studies used metabolomics to reveal potential alterations in metabolites, including lipids, amino acids, carbohydrates, and the tricarboxylic acid (TCA) cycle, in IPF patients or models [7–13]. However, these studies yielded varying results. Additionally, some studies unveiled reverse causality between metabolites and IPF, substantiating the impact of IPF on metabolite alterations. Yet, it remains obscure whether the metabolites or metabolic status in the body affect IPF susceptibility.

Recently, a plethora of genome-wide association studies (GWASs) have amalgamated metabolomics with high-throughput genotyping to evaluate the impact of genetic variants on metabolic traits, ultimately enabling the identification of multiple genetic loci linked to metabolic traits [14]. As a statistical approach reminiscent of randomized controlled trials, Mendelian randomization (MR) utilizes genetic variation to determine whether the association observed between a risk factor and outcomes is indicative of the existence of a causal relationship [15, 16]. Although confounding bias and reverse causality are common limitations in observational studies, they are less likely to appear in MR analyses since genotype formation precedes disease onset [17]. Several MR analyses have confirmed the causal relationship between IPF and exposures such as body mass index (BMI) [18], circulating proteins [19], comorbidities (including gastroesophageal reflux disease (GERD) [20], hypothyroidism [21], venous thromboembolism [22], chronic obstructive pulmonary disease [22], and diabetes [22]).

GWAS has recently been expanded to metabolic phenotyping, generating an atlas of genetically determined metabolites. Considering the uncertainty about the causality between blood metabolites and IPF, the current research assessed the causality between human blood metabolites (486) and the risk of IPF with a bidirectional two-sample MR (TSMR) approach. Further, stable results were selected through meta-analysis combined with sensitivity analysis to identify potential factors influencing IPF.

## Methods

### Study design and data sources

**Fig 1:**
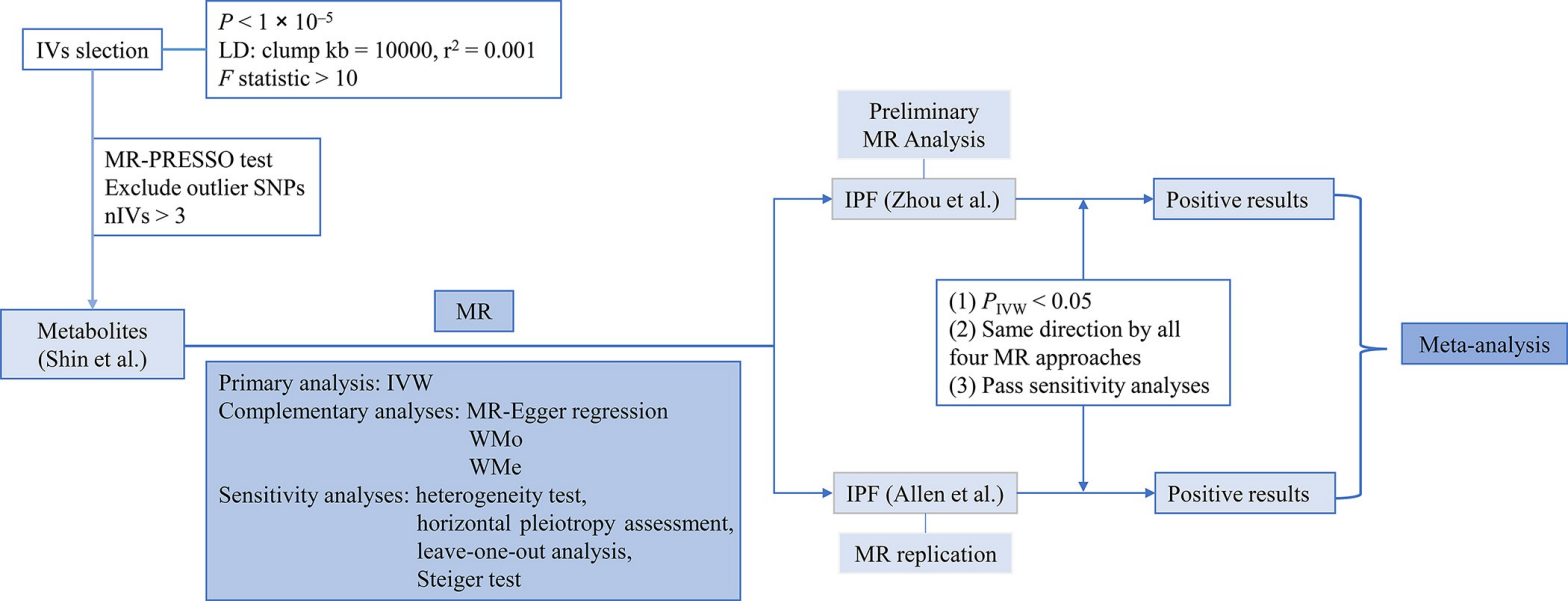
Overview of the current Mendelian randomization study. Reverse MR analysis used similar methods, except that the *P*-value threshold was set to 5 × 10^−6^ for filtering IVs. IV, instrumental variable; nIVs, number of instrumental variables; LD, linkage disequilibrium; MR-PRESSO, MR pleiotropy residual sum, and outlier; IVW, inverse-variance weighted; WMo, weighted mode; WMe, weighted median estimator.

The current research was conducted with a bidirectional TSMR method (Fig 1).

GWAS data on blood metabolites were obtained from a GWAS by Shin et al. which involved 7,824 participants, around 2.1 million single nucleotide polymorphisms (SNPs), and TwinsUK and KORA cohorts (two European cohorts) [14]. The demographic data of both cohorts is detailed in S1 Table. Genetic analysis was carried out with a subset of 486 metabolites (S2 Table), including 309 known metabolites and 177 unknown metabolites [14]. Subsequently, 309 known metabolites were categorized into 8 metabolic clusters (carbohydrates, lipids, amino acids, nucleotides, energy, peptides, vitamins, and xenobiotic metabolism).

The GWAS summary statistics of IPF were extracted from the latest and largest GWAS meta-analysis (947,616 controls and 6,257 IPF cases from 9 biobanks; S3 Table) in the Global Biobank Meta-Analysis Initiative (https://www.globalbiobankmeta.org/resources) developed by Zhou et al. [23]. For the replication analysis, the GWAS data of IPF were acquired from a meta-analysis involving 20,464 controls, 4,125 IPF cases, and 7,554,248 genetic variants [24]. This meta-analysis comprised 5 previous studies of IPF, including UK, Colorado, and Chicago studies, as well as two independent studies (the Genentech study and the USA, UK, and Spain [UUS] study).

### Instrumental variables selection

The genetic variant used as an instrumental variable (IV) should fulfill the following three assumptions [25]: (1) the variant is robustly associated with exposures; (2) the variant is independent of potential confounders; (3) the variant may only affect the outcome through exposures without any direct correlation with the outcome.

For each metabolite assessed, SNPs that exhibited significant associations at a threshold of *P* < 1 × 10^−5^ were selected primarily to maintain an optimal balance between the strength and quantity of IVs [26, 27]. Next, SNPs were clustered by eliminating linkage disequilibrium with R^2^ > 0.001 within 10000 kb in the Phase 3 reference panel of the European 1000 Genomes Project. Exposure-related SNPs were ruled out when they failed to match the obtained outcome GWAS statistics. Palindromic SNPs with an intermediate allele frequency (minor allele frequency > 0.42) were discarded when data were harmonized to align the allele of exposure- and outcome-SNP. *F*-statistics were calculated with the formula in S4 Table, and an *F*-statistic > 10 was used as a threshold for ensuring the power of IVs.

### MR analyses and statistical methods

A TSMR analysis was conducted by including metabolites with more than three independent IVs. First, the MR-pleiotropy residual sum and outlier test (distributions = 1000) was adopted to exclude outlier SNPs [28]. Then, the MR analysis was carried out mainly with the inverse-variance weighted (IVW) method. The consistency of direction was checked with the MR-Egger regression, weighted median estimator (WMe), and weighted mode (WMo).

The Cochran’s Q statistical analysis and MR-Egger intercept test were adopted to test heterogeneity and horizontal pleiotropy, respectively. For positive results, the leave-one-out sensitivity analysis was performed to validate whether single SNPs were involved in the causal relationship in the TSMR analysis (*P* < 0.05 for all but one), followed by the inference of causal direction with the MR Steiger method [29]. To minimize potential confounders, the PhenoScanner database [30] were utilized to identify any associations (*P* = 5 × 10^−8^) with plausible confounders for IVs of positive results.

‘TwoSampleMR 0.5.7’ and ‘MRPRESSO’ in R software-4.3.1 were adopted for TSMR analyses. The code used for TSMR was provided in S1 Appendix. *P*-values were set at less than 0.05 for statistical significance. A meta-analysis was conducted with the Review Manager (version 5.3) random-effects IVW model to determine the robustness of statistically significant results (*P*_IVW_ < 0.05) with consistent direction across the aforementioned four MR methods.

### Mediation analysis

Given that IPF is known to be associated with various factors including BMI, smoking, and GERD, two-step MR for mediation analysis was used to determine whether these factors mediated the effect of metabolites on the risk of IPF. Initially, a TSMR analysis was conducted to ascertain the causal relationship between positive metabolites and BMI, smoking, and GERD, thus verifying the first assumption of MR for mediation analysis, that is, a causal relationship existed between exposures and mediators. The causal relationship between positive metabolites and BMI or GERD was analyzed with the database “ukb-b-19953” or “ebi-a-GCST90000514” [31], and the causal relationship between positive metabolites and smoking was analyzed with a GAWS on lifetime smoking index, which involved 462,690 individuals, from the UK Biobank [32].

Subsequently, IVs were identified for the mediator substances to predict the impact of these mediators on the risk of IPF. If evidence supported that circulating metabolites affect these mediators, which in turn influence IPF, the "product of coefficients" method was utilized to evaluate the indirect effects of circulating metabolites on the risk of IPF through these mediators [33]. The standard errors for these indirect effects were obtained with the delta method [34].

### Reserve TSMR analysis

To determine the impact of IPF on blood metabolites, reverse MR analyses were conducted with similar methods, except that IVs were filtered rigorously according to the threshold of *P* = 5 × 10^−6^.

## Results

### IV selection

S5 and S7 Tables display the characteristics of the genetic IVs used in the TSMR analysis which was performed to clarify the effect of blood metabolites on the risk of IPF. A total of 473 and 476 metabolites were yielded through the TSMR analyses performed with metabolite databases and GWAS datasets from Zhou et al. and Allen et al., respectively, with more than three IVs for each metabolite. The F statistics of all instrumental SNPs were greater than 10, illustrating that IVs were adequately strong.

### Primary MR and sensitivity analyses

In the MR analysis conducted with a metabolite GWAS and an IPF GWAS by Zhou et al. (S6 Table), 21 metabolites were tentatively determined by the IVW method to be substantially related to IPF, among which the chemical compositions of 12 metabolites were identified. Additionally, four MR methods (IVW, WMo, WMe, and MR-Egger regression) revealed that 8 metabolites had a consistent direction (Table 1, S1 Fig). The MR-Egger intercept test demonstrated no horizontal pleiotropy. Meanwhile, no heterogeneity was detected by Cochran’s Q statistical analysis, and no SNP-driven signals were found by the leave-one-out test (S2 Fig).

**Table 1:** MR and sensitivity analysis results of positive metabolites related to IPF.

Metabolites	nSNP	Methods	*P*	OR (95%CI)	Intercept_*P*	Q stat_*P*	Steiger_*P*
**IPF GWAS from Zhou et al.**							
pantothenate	20	IVW	0.044	0.52 (0.27–0.98)	0.844	0.205	< 0.001
guanosine	8	IVW	0.021	1.57 (1.07–2.30)	0.581	0.346	< 0.001
urea	8	IVW	0.004	6.24 (1.77–22.02)	0.349	0.298	< 0.001
serotonin (5HT)	13	IVW	0.004	0.46 (0.27–0.78)	0.826	0.489	< 0.001
glucose	31	IVW	0.024	2.49 (1.13–5.49)	0.328	0.737	< 0.001
caprylate (8:0)	35	IVW	0.026	1.83 (1.08–3.11)	0.720	0.496	< 0.001
ADpSGEGDFXAEGGGVR	5	IVW	0.0496	1.70 (1.00–2.88)	0.743	0.558	< 0.001
4-vinylphenol sulfate	8	IVW	0.015	1.90 (1.13–3.17)	0.576	0.132	< 0.001
**IPF GWAS from Allen et al.**							
guanosine	11	IVW	0.045	1.60 (1.01–2.53)	0.612	0.817	< 0.001
phosphate	5	IVW	0.042	10.37 (1.09–98.60)	0.921	0.246	< 0.001
beta-hydroxyisovalerate	21	IVW	0.042	0.42 (0.18–0.97)	0.850	0.427	< 0.001
heme*	8	IVW	0.033	0.33 (0.12–0.92)	0.726	0.186	< 0.001
HWESASXX*	6	IVW	0.012	0.44 (0.23–0.83)	0.389	0.766	< 0.001
gamma-tocopherol	10	IVW	0.030	1.87 (1.06–3.31)	0.444	0.773	< 0.001
1-palmitoylglycerophosphoinositol*	10	IVW	0.005	2.91 (1.39–6.13)	0.255	0.379	< 0.001
2-tetradecenoyl carnitine	17	IVW	0.012	1.95 (1.16–3.29)	0.249	0.389	< 0.001
4-vinylphenol sulfate	7	IVW	0.008	0.48 (0.28–0.82)	0.687	0.500	< 0.001

IVW, inverse variance weighted; OR, odds ratio; CI, confidence interval; Intercept_*P*, *P* value of the intercept of MR-Egger regression; Q stat_*P*, *P* value of the Cochran’s Q statistical analysis; Steiger_*P*, *P* value of the MR Steiger test.

In the MR analysis performed with GWAS datasets for metabolites and IPF from Allen et al. (S8 Table), 9 metabolites (Table 1) were demonstrated as positive metabolites by the IVW method and had a consistent direction across the four MR methods. The corresponding scatter plots and leave-one-out test results of these nine metabolites are presented in S3 and S4 Figs, respectively. The leave-one-out test for HWESASXX* exhibited that the overall analysis was markedly influenced by the SNP-driven signal in ’rs2007084’. Accordingly, this metabolite was not included as a robust result in the following meta-analysis.

### Meta-analysis

**Fig 2:**
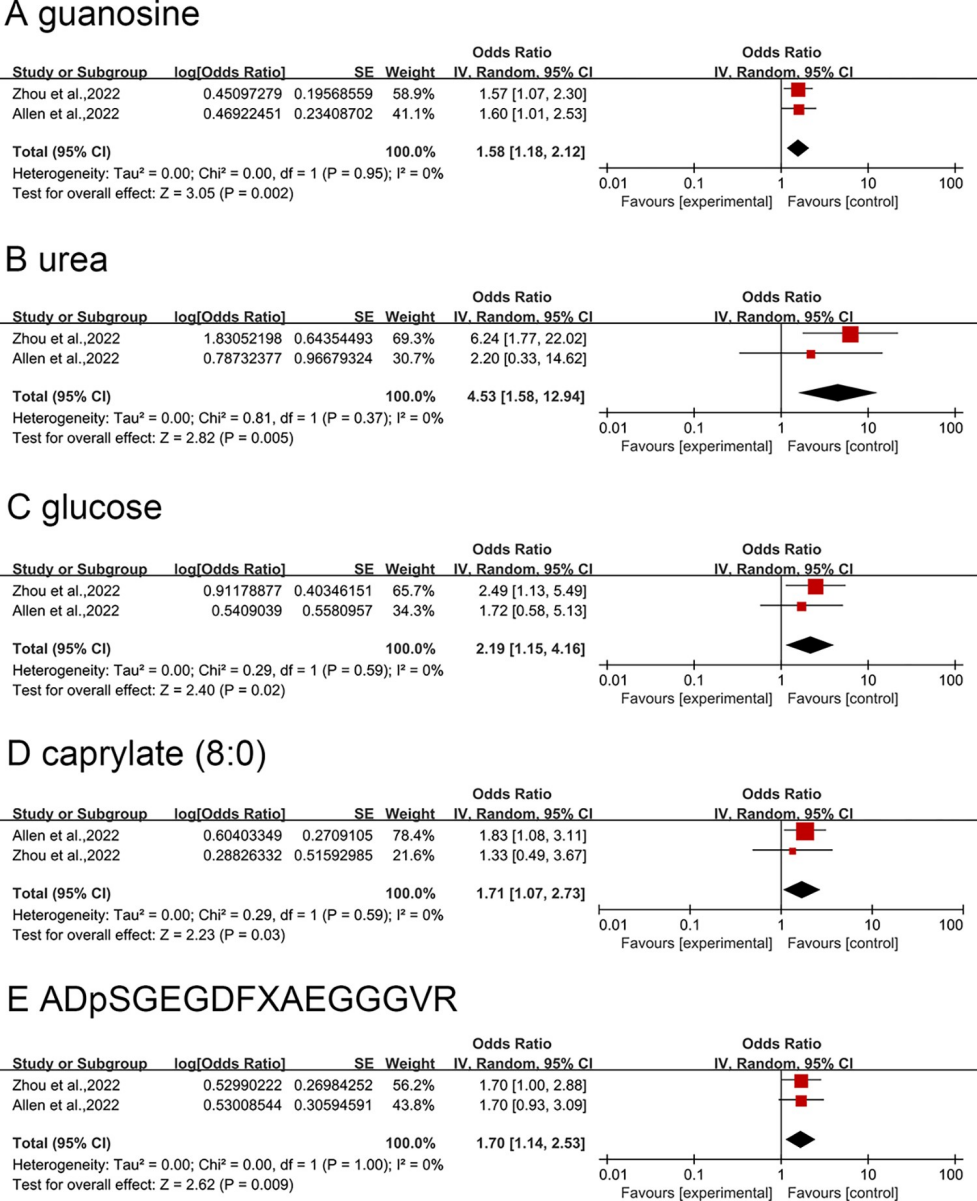
Significant positive causal relationship between metabolites and IPF identified in meta-analysis. The GWAS by Zhou et al.: primary analysis of IPF; the GWAS by Allen et al.: replication analysis of IPF. CI, confidence interval.

A meta-analysis was performed on the 14 robust metabolites obtained with the IPF GWAS data from Zhou et al. and Allen et al. to further validate our results. As depicted in Fig 2, there were marked estimates in the meta-analyses of 5 metabolites. Specifically, susceptibility to IPF was increased by higher levels of guanosine (odds ratio [OR] = 1.58, 95% confidence interval [95%CI] = 1.18–2.12, *P* = 0.002), urea (OR = 4.53, 95%CI = 1.58–12.94, *P* = 0.005), glucose (OR = 2.19, 95%CI = 1.15–4.16, *P* = 0.020), caprylate (8:0) (OR = 1.71, 95%CI = 1.07–2.73, *P* = 0.030), and ADpSGEGDFXAEGGGVR (OR = 1.70, 95%CI = 1.14–2.53, *P* = 0.009). No significant results were yielded for metabolites including pantothenate, serotonin (5HT), 4-vinylphenol sulfate, phosphate, beta-hydroxyisovalerate, heme*, gamma-tocopherol, 1-palmitoylglycerophosphoinositol*, and 2-tetradecenoyl carnitine in the meta-analysis (S5 Fig).

### Confounding analysis

Although sensitivity analyses showed no horizontal pleiotropy for 5 robust metabolites, the secondary traits of metabolite-associated SNPs were further analyzed (S15 Table). With Phenoscanner, one glucose-related SNP (rs17616642) and two caprylate (8:0)-related SNPs (rs1576171 and rs4858696) were excluded due to their correlation with BMI. Subsequent IVW analysis reaffirmed the critical causal relationship between glucose and IPF (OR = 2.68, 95% CI = 1.21−5.96, *P* = 0.016). Conversely, caprylate (8:0) was insignificant correlated with IPF (IVW: OR = 1.59, 95% CI = 0.79−3.22, *P* = 0.194) after BMI-related IVs were excluded.

### Mediation analysis

The preliminary TSMR analysis demonstrated that among the five metabolites with positive results in the meta-analysis, only guanosine exerted a significant positive effect on GERD (IVW: OR = 1.09, 95% CI = 1.00−1.17, *P* = 0.038). Next, the causal effect of GERD on IPF was further assessed. In alignment with the metabolite analysis, IVs for GERD were selected with the *P*-value threshold of 1 × 10^−5^ for analysis, which unraveled that GERD markedly enhanced the risk of IPF (IVW: OR = 1.32, 95% CI = 1.19−1.48, *P* < 0.001). The results of analyses with weighted median and MR-Egger corroborated the assumed directionality in the IVW MR analysis. Finally, it was observed that the causal effect of guanosine on IPF via GERD was 0.023 (95%CI = 0.001−0.049), with a mediation proportion of 5% (Table 2).

**Table 2:** The mediation effect of guanosine on IPF via GERD.

Mediator	Total effect	Direct effect A	Direct effect B	Mediation effect	Mediation proportion (%)
	Beta (95%CI)	Beta (95%CI)	Beta (95%CI)	Beta (95%CI)	
GERD	0.451 (0.067, 0.835)	0.082 (0.005, 0.159)	0.280 (0.171, 0.390)	0.023 (0.001, 0.049)	5

Note: “Total effect” indicates the effect of guanosine on IPF. “Direct effect A” represents the effect of guanosine on GERD, and “direct effect B” stands for the effect of GERD on IPF. “Mediation effect” indicates the effect of guanosine on IPF via GERD. Total and direct effects (A and B) were estimated with IVW, and the mediation effect was calculated with the delta method.

### Reserve TSMR analysis

The reserve TSMR analysis was utilized to delve into the effect of IPF on blood metabolites, with *P* < 5 × 10^−6^ as a screening threshold of IVs for IPF. S9–S11 Tables respectively display the impact of IPF on blood metabolites in the MR analysis conducted with IPF GWAS data from Zhou et al., the results of the sensitivity analysis, and the characteristics of the used SNPs. Additionally, S12–S14 Tables list the results obtained with IPF GWAS data from Allen et al.

A total of 15 positive metabolites were identified through the preliminary screening (S6 Fig), among which 6 metabolites, including leucine, linoleate (18:2n6), guanosine, laurate (12:0), 3-carboxy-4-methyl-5-propyl-2-furanpropanoate (CMPF), and 1,7-dimethylurate, still showed positive results in meta-analysis. Nevertheless, ORs were extremely close to 1 in the reverse MR analysis, highlighting a weak impact of IPF on blood metabolites.

## Discussion

In the current research, a TSMR analysis was carried out to identify the causality between blood metabolites (486) and IPF. The initial MR analysis was conducted with the IPF database from Zhou et al., while the replication analysis was performed with the database from Allen et al. In preliminary and replicated MR analyses, 8 and 9 positive metabolites were obtained, respectively. After excluding duplicates and those that failed the sensitivity analysis, a total of 14 metabolites were included in the meta-analysis. Ultimately, 5 metabolites had positive results in the meta-analysis. To be specific, higher levels of guanosine, urea, glucose, caprylate (8:0), and ADpSGEGDFXAEGGGVR shared a causal association with the elevated risk of IPF.

For these 5 positive metabolites, the secondary traits of metabolite-associated SNPs were further analyzed with Phenoscanner. After BMI-related SNPs were excluded, glucose was still significantly correlated with IPF, as opposed to caprylate (8:0). Therefore, the positive effect of caprylate (8:0) on IPF in the preliminary MR analysis might be confounded by BMI, which was excluded from the robust results. In addition, the results of MR for mediation analysis revealed that the promoting effect of guanosine on IPF was partly mediated by GERD, with a mediation proportion of 5%. Reverse MR analysis results exhibited that IPF might also exert a slightly elevating effect on guanosine levels in the blood.

Recently, metabolomic research has extensively displayed the perturbation of metabolic pathways, such as lipids, amino acids, carbohydrates, and the TCA cycle, during IPF [35]. Notably, mounting studies have reported the pivotal involvement of glycolytic reprogramming in IPF pathogenesis. For instance, the research by Xie et al. [9] elucidated that increased glycolysis was an early and sustained event during myoblast differentiation and that pharmacological or genetic interventions repressed the glycolytic enzyme 6-phosphofructo-2-kinase/fructose-2, 6-biphosphatase 3 and diminished fibroblast activation in vitro, as well as substantially ameliorating PF in mice [9]. Compared to oxidative phosphorylation, glycolysis can produce higher levels of lactate, which modulates histone modification, macrophage proliferation, and fibroproliferation, thereby perpetuating fibrosis [36, 37]. A recent study revealed that glucose transporters (GLUTs) were amplified during the progression of fibrogenesis and strongly emphasized the profibrotic role of upregulated GLUT1 and subsequently increased glucose uptake [38]. Hyperglycemia has been confirmed to provide more prerequisites for glycolysis. Nonetheless, lactic acid levels exerted an insignificant effect on IPF in our study. Accordingly, further research is warranted to discuss whether hyperglycemia drives PF by enhancing glycolysis.

The relationship between diabetes and IPF has been controversial. For example, a prior meta-analysis, which involved nine case–control studies with 19,095 control subjects and 5,096 IPF patients, exhibited a positive association of diabetes with IPF (OR = 1.65, 95%CI = 1.30–2.10, *P* < 0.0001) [39]. However, a prior MR study demonstrated no discernible causal relationship between diabetes and IPF [22]. When the relationship between diabetes and IPF was explored, potential biases stemming from blood glucose management in diabetic patients may account for heterogeneity in outcomes. The phenotype of blood glucose can be used to provide a more direct view of the relationship between diabetes and IPF. Therefore, more clinical studies are needed to focus on the relationship between blood glucose and the onset or prognosis of IPF, not solely on the diagnosis of diabetes.

A multitude of studies have evidenced the alleviatory effect of the hypoglycemic drug metformin on IPF in mice [40–44]. Intriguingly, the effectiveness of metformin in the treatment of IPF varies across clinical trials. For instance, a post hoc analysis unraveled no substantial difference in clinical efficacy between the combination of pirfenidone and metformin and pirfenidone monotherapy, irrespective of diabetes in IPF patients [45]. On the contrary, a national cohort study revealed that metformin had clinical advantages in IPF patients with diabetes since it reduced overall mortality and hospitalization rates [46]. Considering our result that hyperglycemia served as a factor elevating the risk of IPF, blood glucose management may assume a role in lowering IPF prevalence, and more clinical trials are needed to validate this result.

In addition, the present study also showed that the elevation in blood urea, guanosine, and ADpSGEGDFXAEGGGVR was associated with an increased risk of IPF. Urea synthesis is an imperative process of the ornithine cycle in the liver and functions as the paramount conduit for the management of ammonia metabolism. Arginine is a key intermediate compound in the ornithine cycle, and its metabolite proline is essential in promoting collagen accumulation during fibrosis [47]. The study by Zhao et al. [11] unveiled that the metabolites of arginine, including creatine, proline, putrescine, and spermidine, were upregulated in the lung tissues of IPF patients. Similarly, our reverse MR analysis result also displayed an effect of IPF in upregulating proline, but the significance of this effect was diminished in the meta-analysis. In addition, arginase-1, an enzyme implicated in the cleavage of arginine to generate proline, ornithine, and urea, was found to be upregulated in models of bleomycin-stimulated PF [48]. In summary, the MR analysis exhibited the promoting effect of high urea levels on IPF, illustrating the potential involvement of ornithine cycle disturbance in IPF. Limited research has been performed on the association of guanosine and ADpSGEGDFXAEGGGVR with IPF. Our data elucidated that the upregulation of ADpSGEGDFXAEGGGVR conferred a slightly elevating effect on IPF risk, with a *P*-value (0.0496) at the edge of the threshold value.

Several limitations exist in our study. First, it is imperative to be cautious in interpreting and extrapolating the results of MR studies [49]. Further external validation should be performed to confirm the results. Second, only GWAS data on individuals of European ancestry were employed to minimize the source of variation. Accordingly, the generalization of our results to other populations remains uncertain, which necessitates further exploration and validation in diverse ethnic backgrounds. Third, the accuracy and reliability of MR analysis results are contingent on the quality and sample size of the database. Larger datasets can minimize potential biases and enhance the robustness of our conclusions.

### Conclusion

In conclusion, our MR results identified 4 metabolites that might elevate IPF risk, including glucose, urea, guanosine, and ADpSGEGDFXAEGGGVR. Our results provide clues to mechanisms behind the occurrence and development of IPF and may have implications for its treatment.

## Supporting information

S1 Fig Scatter plots for 8 metabolites with positive meta-analysis results from primary reserve MR analysis using metabolites GWAS and IPF GWAS by Zhou et al. (TIF)

S2 Fig Leave-one-out plots for 8 metabolites with positive meta-analysis results from primary reserve MR analysis using metabolites GWAS and IPF GWAS by Zhou et al. (TIF)

S3 Fig Scatter plots for 9 metabolites with positive meta-analysis results from primary reserve MR analysis using metabolites GWAS and IPF GWAS by Allen et al. (TIF)

S4 Fig Leave-one-out plots for 9 metabolites with positive meta-analysis results from primary reserve MR analysis using metabolites GWAS and IPF GWAS by Allen et al. (TIF)

S5 Fig Negative causal links between metabolites and IPF after meta-analysis. (TIF)

S6 Fig Meta-analysis of the causal associations of IPF on metabolites. (TIF)

S1 Table Summary information for the cohorts of blood metabolites GWAS. (XLSX)

S2 Table Metabolite information. (XLSX)

S3 Table The composition of IPF GWAS from Zhou et al. (XLSX)

S4 Table The formula used to calculate R2 and F statistic between exposure and outcome. (XLSX)

S5 Table Characteristics of the genetic IVs used in TSMR analysis for the effect of blood metabolites on IPF risk (*P* < 1.00E-05, IPF GWAS from Zhou et al.). (XLSX)

S6 Table Effect of blood metabolites on IPF risk (IPF GWAS from Zhou et al.). (XLSX)

S7 Table Characteristics of the genetic IVs used in TSMR analysis for the effect of blood metabolites on IPF risk (*P* < 1.00E-05, IPF GWAS from Allen et al.). (XLSX)

S8 Table Effect of blood metabolites on IPF risk (IPF GWAS from Allen et al.). (XLSX)

S9 Table Effect of IPF on blood metabolites (*P* < 5.00E-06, IPF GWAS from Zhou et al.). (XLSX)

S10 Table Sensitivity analysis results of the MR analyses for the effect of IPF on blood metabolites (*P* < 5.00E-06, IPF GWAS from Zhou et al.). (XLSX)

S11 Table Characteristics of the genetic IVs used in TSMR analysis for the effect of IPF on blood metabolites (*P* < 5.00E-06, IPF GWAS from Zhou et al.). (XLSX)

S12 Table Effect of IPF on blood metabolites (*P* < 5.00E-06, IPF GWAS from Allen et al.). (XLSX)

S13 Table Sensitivity analysis results of the MR analyses for the effect of IPF on blood metabolites (*P* < 5.00E-06, IPF GWAS from Allen et al.). (XLSX)

S14 Table Characteristics of the genetic IVs used in TSMR analysis for the effect of IPF on blood metabolites (*P* < 5.00E-06, IPF GWAS from Allen et al.). (XLSX)

S15 Table Results of the query in PhenoScanner for positive metabolites after meta-analysis. (XLSX)

S1 Appendix Code. (DOCX)

## Abbreviations

IPF: Idiopathic pulmonary fibrosis
TCA: Tricarboxylic acid
GWAS: Genome-wide association study
MR: Mendelian randomization
BMI: Body mass index
GERD: Gastroesophageal reflux disease
TSMR: Two-sample Mendelian randomization
SNP: Single nucleotide polymorphisms
IV: Instrumental variable
IVW: Inverse-variance weighted
WMe: Weighted median estimator
WMo: Weighted mode
GLUT: Glucose transporter
