# Supplementary figures and images for: The causal relationship between genetically predicted blood metabolites and idiopathic pulmonary fibrosis: A bidirectional two-sample Mendelian randomization study
Source: PLoS One. 2024 Apr 16;19(4):e0300423. doi: 10.1371/journal.pone.0300423 (PMC11020755; doi:10.1371/journal.pone.0300423)

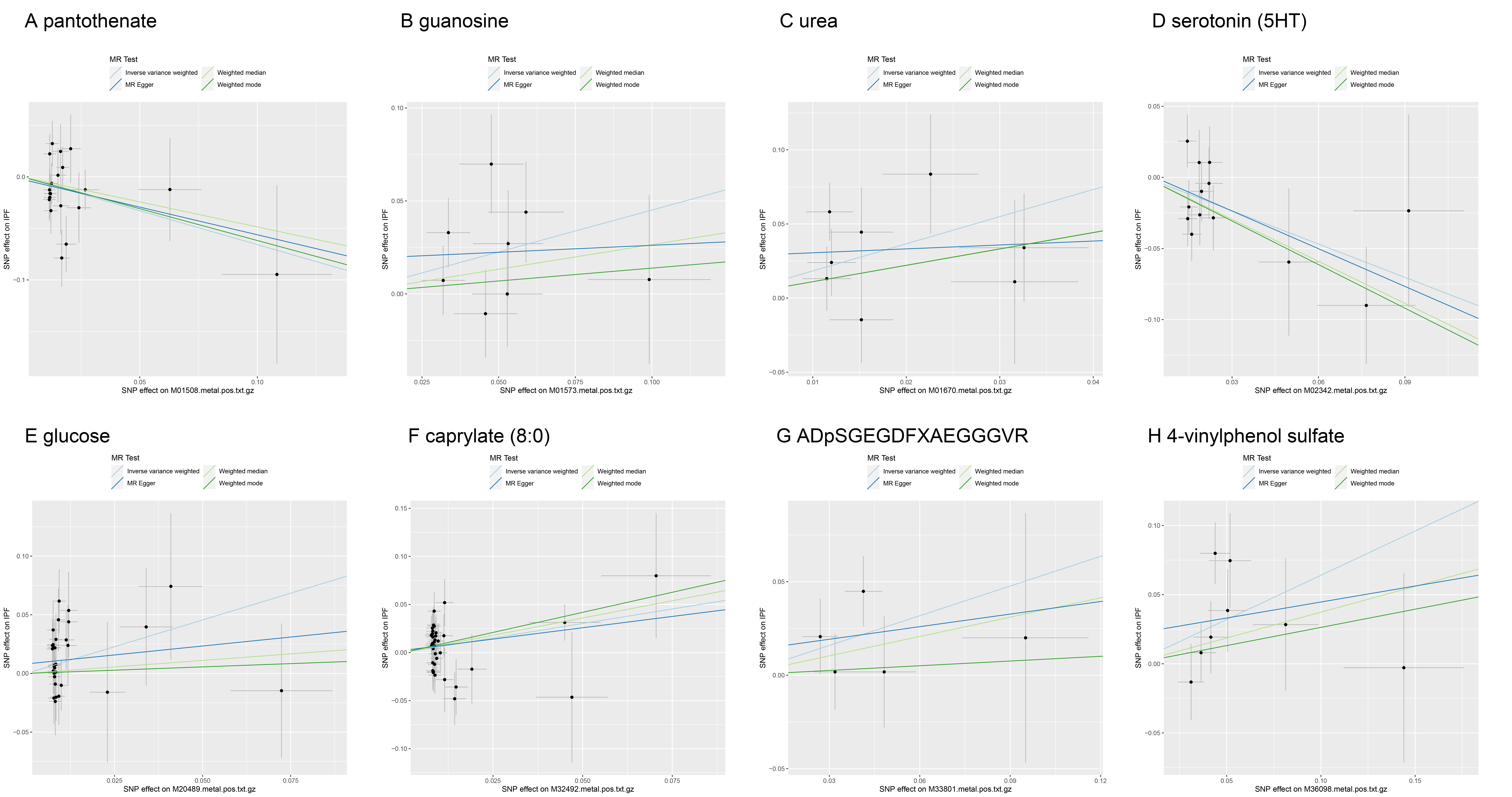

Supplement: S1 Fig — (TIF) [file pone.0300423.s001.tif]

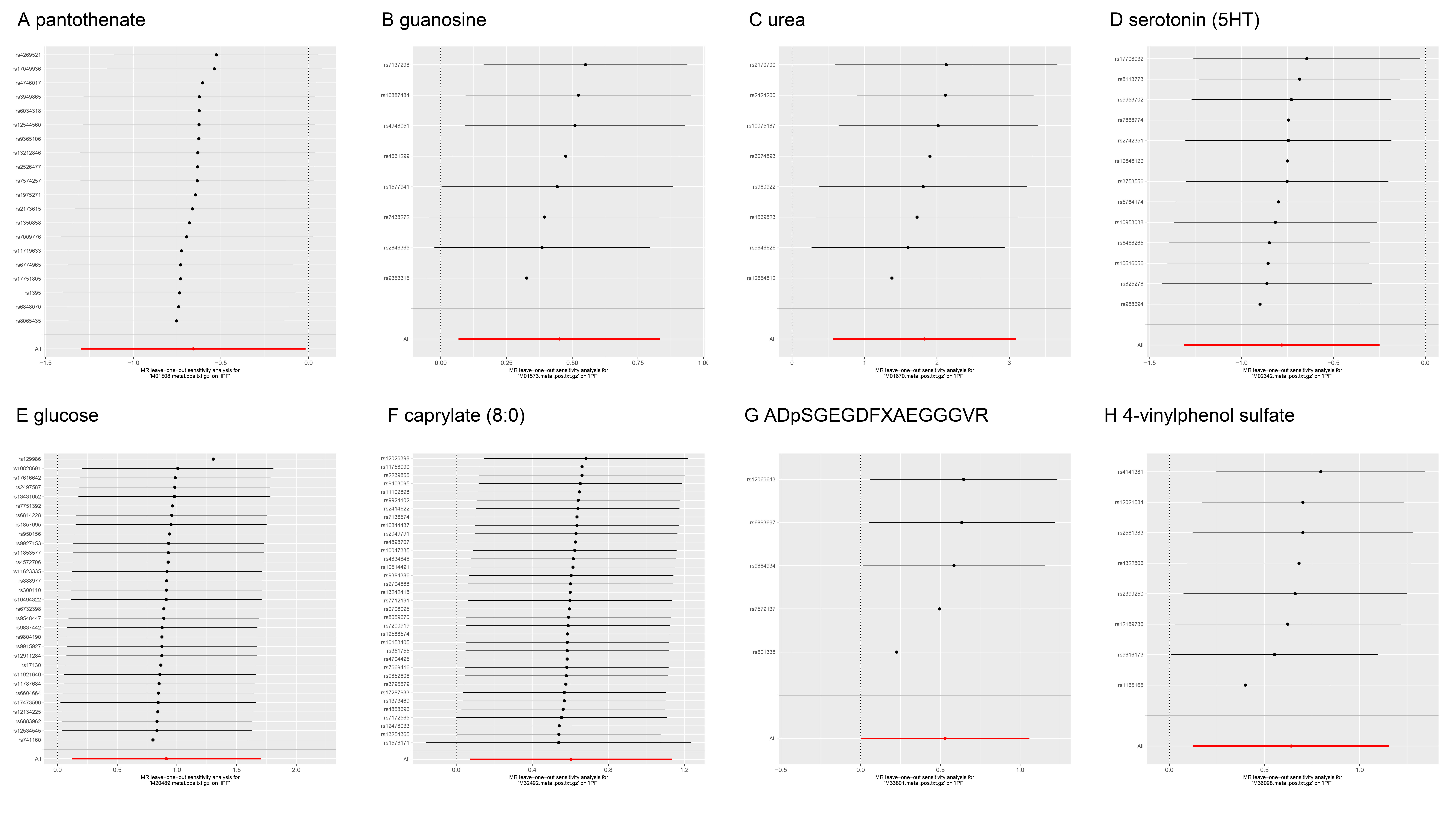

Supplement: S2 Fig — (TIF) [file pone.0300423.s002.tif]

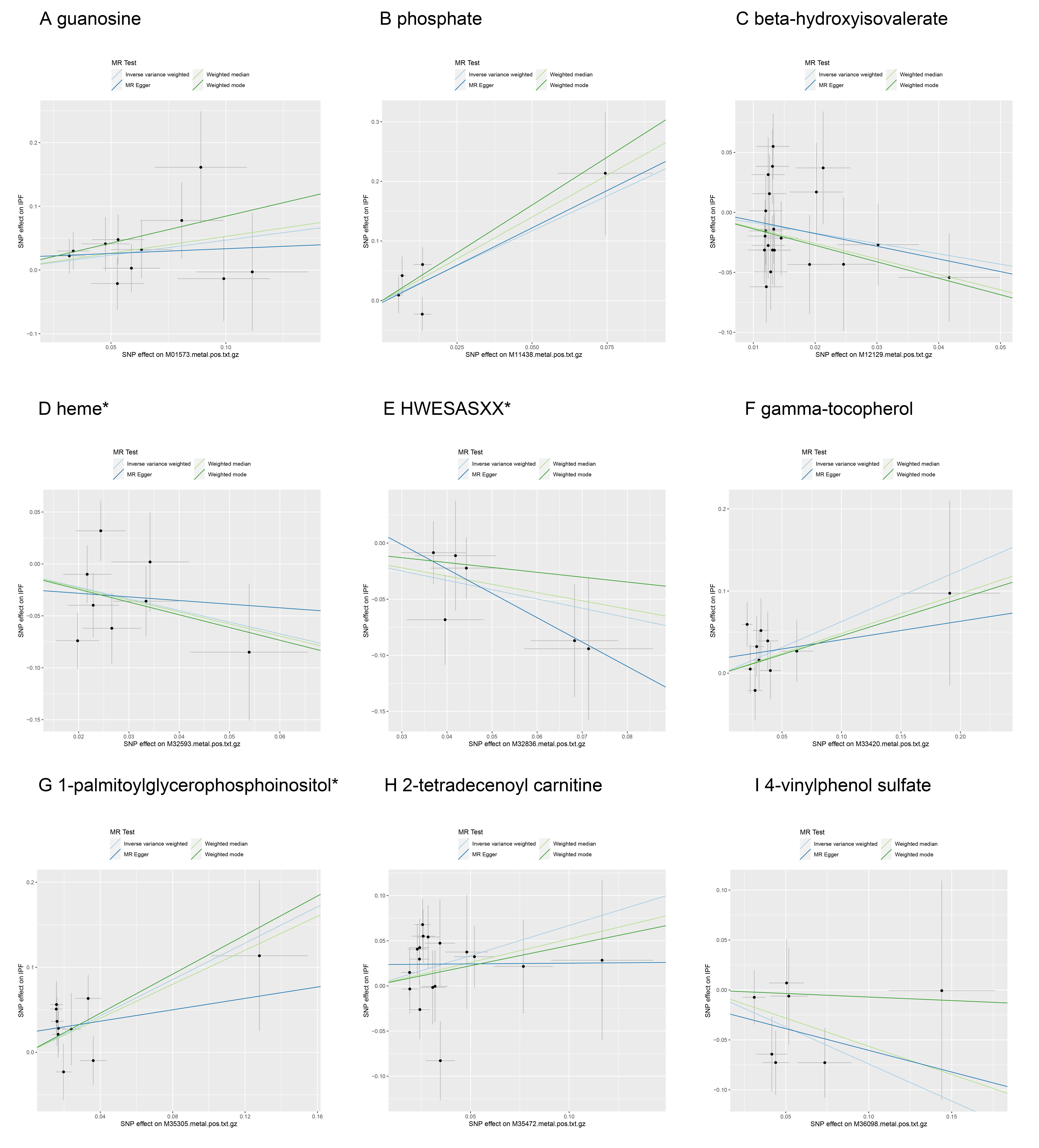

Supplement: S3 Fig — (TIF) [file pone.0300423.s003.tif]

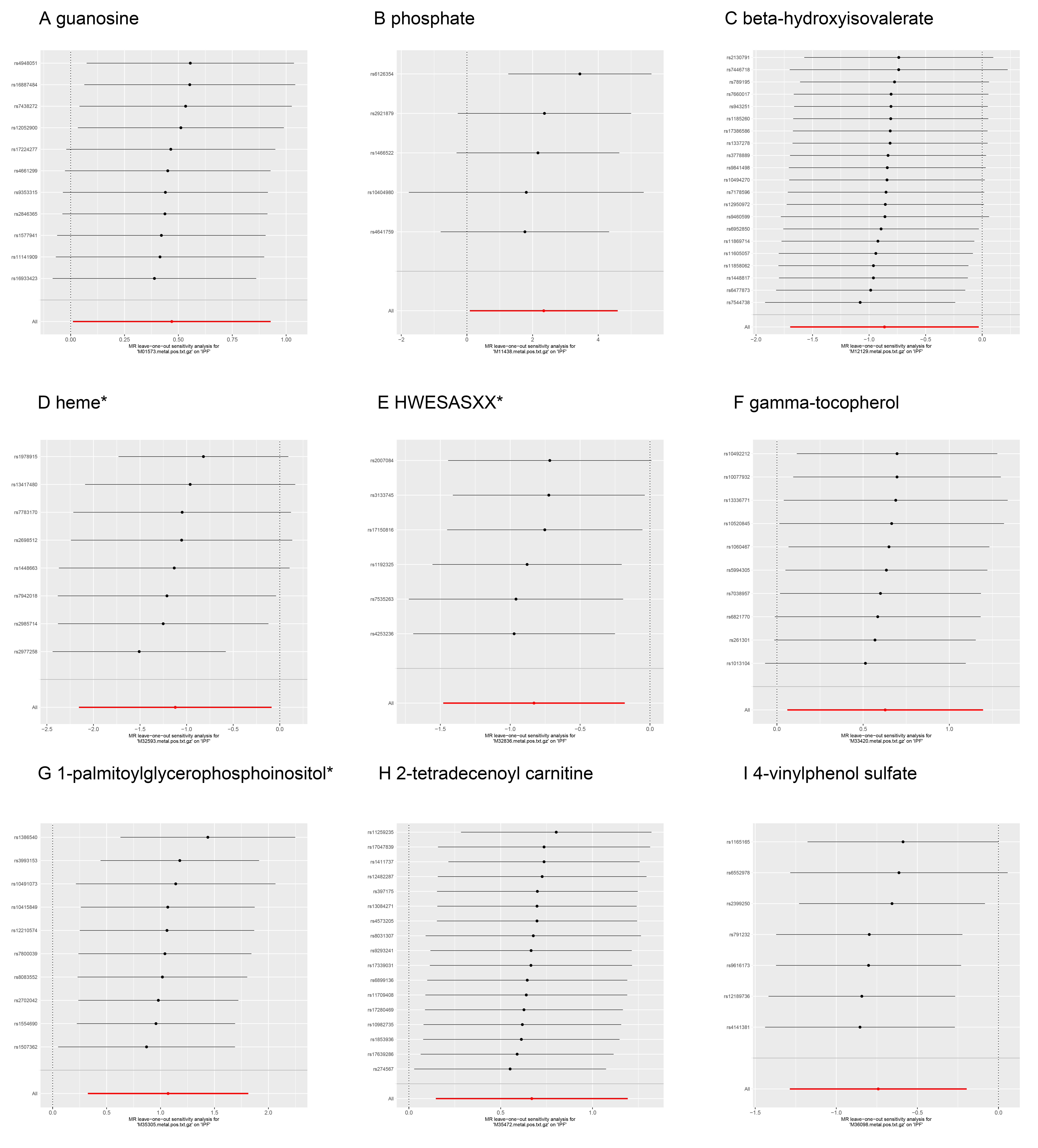

Supplement: S4 Fig — (TIF) [file pone.0300423.s004.tif]

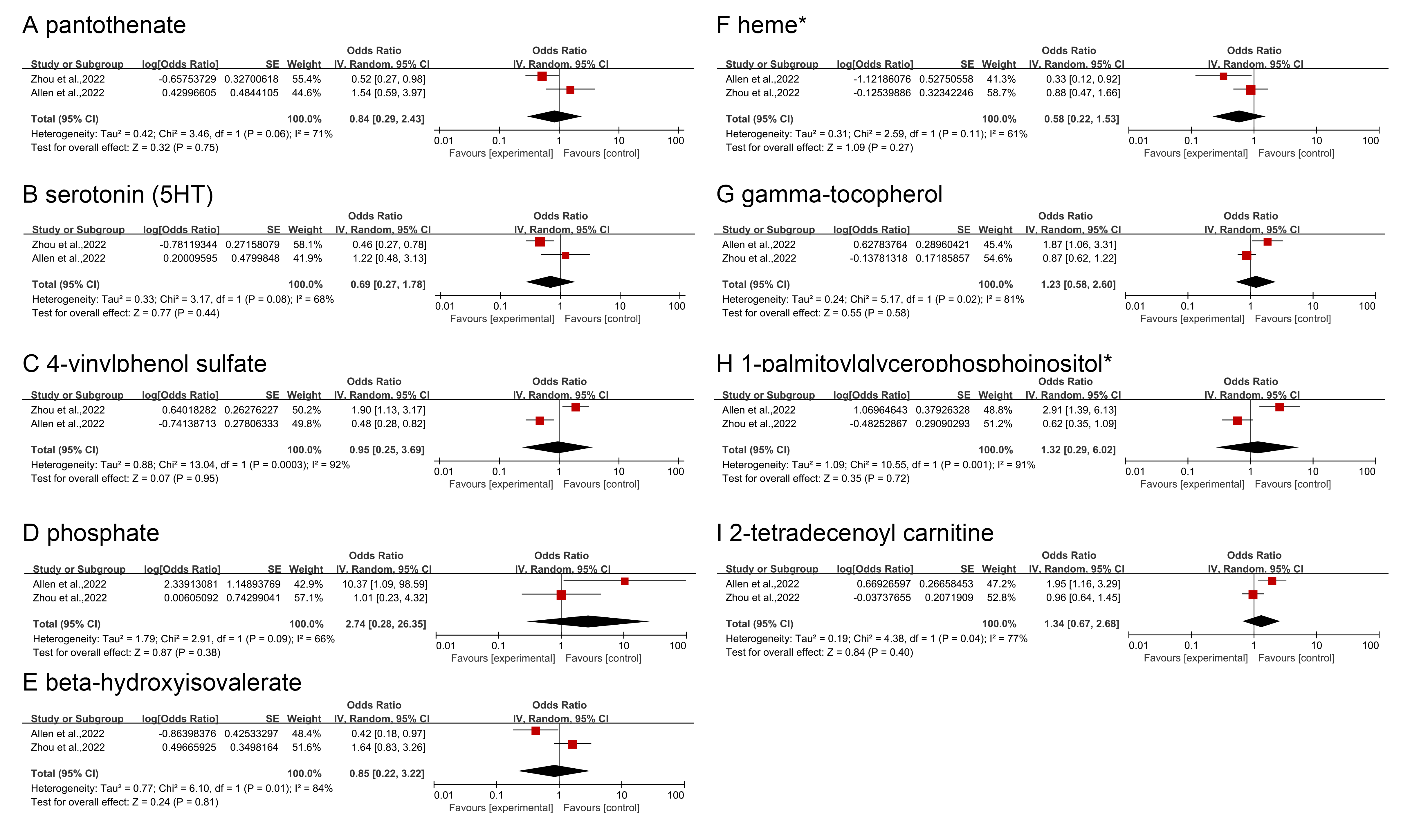

Supplement: S5 Fig — (TIF) [file pone.0300423.s005.tif]

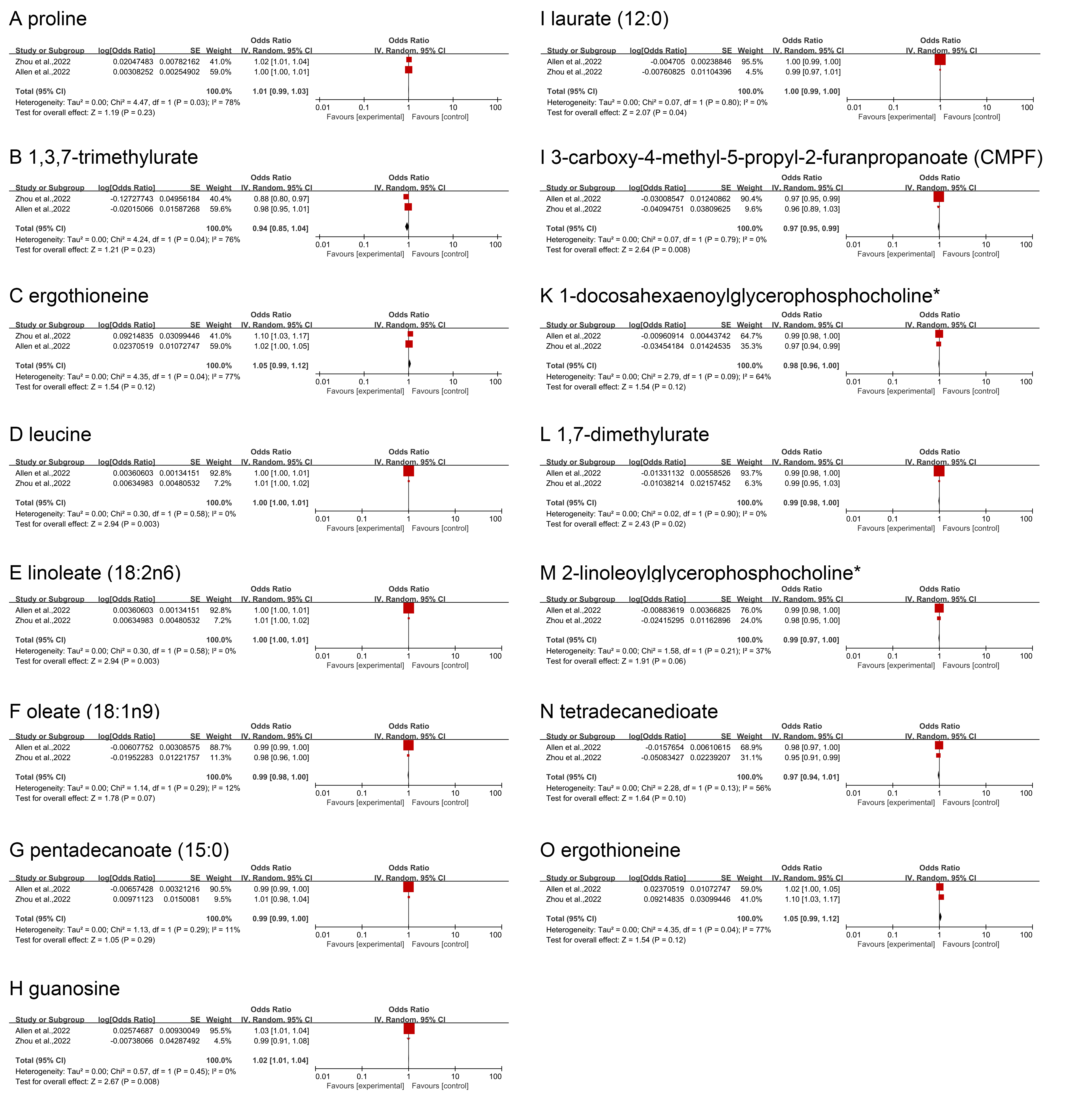

Supplement: S6 Fig — (TIF) [file pone.0300423.s006.tif]
