# Supplementary material for: The causal relationship between genetically predicted blood metabolites and idiopathic pulmonary fibrosis: A bidirectional two-sample Mendelian randomization study
Source: PLoS One. 2024 Apr 16;19(4):e0300423. doi: 10.1371/journal.pone.0300423 (PMC11020755; doi:10.1371/journal.pone.0300423)
Supplement: S1 Appendix — (DOCX) [file pone.0300423.s022.docx]

TSMR loop code

#install.packages("R.utils")

library(TwoSampleMR)

library(data.table)

library(tidyverse)

library(readxl)

library(writexl)

library(ieugwasr)

#

FileNames <-list.files(paste0(getwd()),pattern=".gz")

exp_dat_ids <- FileNames

exps <- FileNames

#

out<-fread(file = 'D:/R/IPF EUR/IPF_Bothsex_eur_inv_var_meta_GBMI_052021_nbbkgt1.txt',

header = T)

colnames(out) <- c("chrom","pos","other_allele","effect_allele","SNP","eaf","beta","se","pval","inv_var_het_p","direction","N_case","N_ctrl","n_dataset","n_bbk","is_strand_flip","is_diff_AF_gnomAD")

out$trait <- 'IPF'

out <- mutate(out, SampleSize = N_case+N_ctrl)

head(out)

outcomeid <- out

rm(out)

head(outcomeid)

#ALLEN

out<-fread(file = 'D:/R//meta_gwas_5way_summary_stats 2022 Allen.txt', sep = ' ', header = T, check.names = F)

colnames(out) <- c("chromosome","position","SNP","other_allele","effect_allele","maf","studies","n","Direction","beta","se","pval")

out$trait <- 'IPF'

out <- mutate(out, SampleSize = 24589)

head(out)

outcomeid <- out

rm(out)

head(outcomeid)

snp_add_eaf <- function(dat, build = "37", pop = "EUR")

{

stopifnot(build %in% c("37","38"))

stopifnot("SNP" %in% names(dat))

# Create and get a url

server <- ifelse(build == "37","http://grch37.rest.ensembl.org","http://rest.ensembl.org")

pop <- paste0("1000GENOMES:phase_3:",pop)

snp_reverse_base <- function(x)

{

x <- stringr::str_to_upper(x)

stopifnot(x %in% c("A","T","C","G"))

switch(x,"A"="T","T"="A","C"="G","G"="C")

}

res_tab <- lapply(1:nrow(dat), function(i)

{

print(paste0("seaching for No.", i, " SNP"))

dat_i <- dat[i,]

ext <- paste0("/variation/Homo_sapiens/",dat_i$SNP, "?content-type=application/json;pops=1")

url <- paste(server, ext, sep = "")

res <- httr::GET(url)

# Converts http errors to R errors or warnings

httr::stop_for_status(res)

# Convert R objects from JSON

res <- httr::content(res)

res_pop <- jsonlite::fromJSON(jsonlite::toJSON(res))$populations

# Filter query results based on population set

res_pop <- try(res_pop[res_pop$population == pop,])

if("try-error" %in% class(res_pop))

{

print(paste0("There is not information for population ",pop))

queried_effect_allele <- "NR"

queried_other_allele <- "NR"

queried_eaf <- -1

}

else

{

if(nrow(res_pop)==0)

{

print(paste0("There is not information for population ",pop))

queried_effect_allele <- "NR"

queried_other_allele <- "NR"

queried_eaf <- -1

}

else

{

queried_effect_allele <- res_pop[1,"allele"][[1]]

queried_other_allele <- res_pop[2,"allele"][[1]]

queried_eaf <- res_pop[1,"frequency"][[1]]

}

}

effect_allele <- ifelse("effect_allele.exposure" %in% names(dat),

dat_i$effect_allele.exposure,

dat_i$effect_allele)

other_allele <- ifelse("effect_allele.exposure" %in% names(dat),

dat_i$other_allele.exposure,

dat_i$other_allele)

if("effect_allele.exposure" %in% names(dat))

{

name_output <- unique(c(names(dat), "eaf.exposure","reliability.exposure"))

}

else

{

name_output <- unique(c(names(dat), "eaf","reliability.exposure"))

}

len_effect_allele <- nchar(effect_allele)

len_other_allele <- nchar(other_allele)

if(len_effect_allele==1&len_other_allele==1)

{

if((queried_effect_allele==effect_allele & queried_other_allele==other_allele)|

(queried_effect_allele==other_allele & queried_other_allele==effect_allele))

{

dat_i$eaf.exposure <- ifelse(effect_allele == queried_effect_allele,

queried_eaf,

1-queried_eaf)

dat_i$eaf <- dat_i$eaf.exposure

dat_i$reliability.exposure <- "high"

}

else

{

r_queried_effect_allele <- snp_reverse_base(queried_effect_allele)

r_queried_other_allele <- snp_reverse_base(queried_other_allele)

if((r_queried_effect_allele==effect_allele & r_queried_other_allele==other_allele)|

(r_queried_effect_allele==other_allele & r_queried_other_allele==effect_allele))

{

dat_i$eaf.exposure <- ifelse(effect_allele == r_queried_effect_allele,

queried_eaf,

1-queried_eaf)

dat_i$eaf <- dat_i$eaf.exposure

dat_i$reliability.exposure <- "high"

}

else

{

dat_i$eaf.exposure <- ifelse(effect_allele == queried_effect_allele,

queried_eaf,

1-queried_eaf)

dat_i$eaf <- dat_i$eaf.exposure

dat_i$reliability.exposure <- "low"

}

}

}

else

{

# To identify the potential DEL/ INS

short_allele <- ifelse(len_effect_allele==1,

effect_allele,

other_allele)

short_allele_eaf <- ifelse(short_allele == queried_effect_allele,

queried_eaf,

1-queried_eaf)

dat_i$eaf.exposure <- ifelse(effect_allele == short_allele,

short_allele_eaf,

1-short_allele_eaf)

dat_i$eaf <- dat_i$eaf.exposure

dat_i$reliability.exposure <- "low"

}

dat_i[name_output]

})

return(do.call(rbind, res_tab))

}

#

dir.create(path = "Filename")

#######cyclic code######################

qaq <- 1

for (qaq in 1:length(exp_dat_ids)) { #

exp_dat_id <- exp_dat_ids[qaq]

exp <- exps[qaq]

d3<- try(fread(paste0(getwd(),"/",FileNames[qaq]),fill=TRUE),silent = T)

d3<-subset(d3,d3$`P-value`<1e-5)

# rm(d1)

# d3<-d2[,c(1,2,3,4,8,9,10,16)]

d3$PHENO <- FileNames[qaq]

names(d3)[names(d3) == 'MarkerName'] <- 'SNP'

#SNPS associated with confounding factors were removed

d3<-d3[!(d3$SNP %in% c("SNP A","SNP B")),]

d3<-format_data(d3,

type="exposure",

phenotype_col = "PHENO",

snp_col = "SNP",

beta_col = "Effect",

se_col = "StdErr",

pval_col = "P-value",

samplesize_col = "TotalSampleSize",

eaf_col = "Freq1",

effect_allele_col = "Allele1",

other_allele_col = "Allele2")

#exp_data <- clump_data(d3,clump_kb = 500,clump_r2 = 0.01)

d4<- ld_clump(

#dat = X1,

clump_kb = 10000,

clump_r2 = 0.001,

pop = "EUR",

dplyr::tibble(rsid=d3$SNP, pval=d3$pval.exposure, id=d3$id.exposure),

#get_plink_exe()

plink_bin = "C:/Users/lx/Desktop/plink_win64_20230116/plink.exe",

bfile = "C:/Users/lx/Desktop/1kg.v3.tar/1kg.v3/EUR"

)

exp_data<-subset(d3,SNP %in% d4$rsid)

if(length(exp_data[,1])>0){

outcome_dat<-merge(exp_data,outcomeid,by.x = "SNP",by.y = "SNP")

write.csv(outcome_dat,file = "d.csv")

out_data <- read_outcome_data(

snps = exp_data$SNP,

filename = "d.csv",

sep = ",",

phenotype_col = "trait",

snp_col = "SNP",

beta_col = "beta",

se_col = "se",

eaf_col="eaf",

samplesize_col = "SampleSize",

effect_allele_col = "effect_allele",

other_allele_col = "other_allele",

pval_col = "pval")

#add eaf

# write.table(out_data,file ="out_data")

# out_data<-snp_add_eaf(out_data)

if(length(out_data[,1])>0){

dat <- TwoSampleMR::harmonise_data(

exposure_dat = exp_data,

outcome_dat = out_data)

####

dat <-subset(dat,mr_keep==TRUE)

#F-statistic and R2

get_f<-function(dat,F_value=10){

log<-is.na(dat$eaf.exposure)

log<-unique(log)

if(length(log)==1)

{if(log==TRUE){

print("no eaf, so the F statistic cannot be calculated")

return(dat)}

}

if(is.null(dat$beta.exposure[1])==T || is.na(dat$beta.exposure[1])==T){print("no eaf, so the F statistic cannot be calculated")

return(dat)}

if(is.null(dat$se.exposure[1])==T || is.na(dat$se.exposure[1])==T){print("no se, so the F statistic cannot be calculated")

return(dat)}

if(is.null(dat$samplesize.exposure[1])==T || is.na(dat$samplesize.exposure[1])==T){print("no samplesize, so the F statistic cannot be calculated")

return(dat)}

if("FALSE"%in%log && is.null(dat$beta.exposure[1])==F && is.na(dat$beta.exposure[1])==F && is.null(dat$se.exposure[1])==F && is.na(dat$se.exposure[1])==F && is.null(dat$samplesize.exposure[1])==F && is.na(dat$samplesize.exposure[1])==F){

R2<-(2*(1-dat$eaf.exposure)*dat$eaf.exposure*(dat$beta.exposure^2))/((2*(1-dat$eaf.exposure)*dat$eaf.exposure*(dat$beta.exposure^2))+(2*(1-dat$eaf.exposure)*dat$eaf.exposure*(dat$se.exposure^2)*dat$samplesize.exposure))

F<- (dat$samplesize.exposure-2)*R2/(1-R2)

dat$R2<-R2

dat$F<-F

dat<-subset(dat,F>F_value)

return(dat)

}

}

dat <- get_f(dat, F_value = 10)

#####MR-PRESSO######

# PRESSO

mr_Presso<-function(dat,num=10000){

library(TwoSampleMR)

library(MRPRESSO)

library(dplyr)

set.seed(123)

try (mr_presso_res<-mr_presso(BetaOutcome ="beta.outcome", BetaExposure = "beta.exposure", SdOutcome ="se.outcome", SdExposure = "se.exposure",

OUTLIERtest = TRUE,DISTORTIONtest = TRUE, data = dat,

SignifThreshold = 0.05, NbDistribution = num))

return(mr_presso_res)

}

mr_presso_pval<-function(mr_presso_res){

try ( mr_presso_main<-mr_presso_res$`Main MR results`)

try ( mr_presso_main[3:5,]<-NA)

return(mr_presso_main)

}

mr_presso_snp<-function(mr_presso_res,mr_presso_main,dat,type="list"){

data_re<-list()

if(type=="list"){

for(i in 1:length(mr_presso_res)){

res<-mr_presso_res[[i]]

main<-mr_presso_main[[i]]

data<-dat[[i]]

try(if(is.na(main[2,6])==FALSE){

outliers<-which(res$`Outlier Test`$Pvalue<0.05)

data$mr_keep[outliers]<-FALSE

})

data_re[[i]]<-data

names(data_re)[[i]]<-names(dat)[[i]]

}

return(data_re)

}

if(type=="data"){

res<-mr_presso_res$`MR-PRESSO results`

main<-mr_presso_main

data<-dat

try(if(is.na(main[2,6])==FALSE){

outliers<-which(res$`Outlier Test`$Pvalue<0.05)

data$mr_keep[outliers]<-FALSE

})

return(data)

}

}

#MR-PRESSO （find outliers）

mr_presso_res <- mr_Presso(dat, num = 1000)

mr_presso_main <- mr_presso_pval(mr_presso_res)

dat <- mr_presso_snp(mr_presso_res, mr_presso_main, dat, type = "data")

###se is calculated according to beta and p

resMRPRESSO=mr_presso_res[["Main MR results"]]

resMRPRESSO

global_test_p <- mr_presso_res[["MR-PRESSO results"]][["Global Test"]][["Pvalue"]]

se1=sqrt(((resMRPRESSO[1,3])^2)/qchisq(Pval_raw <- resMRPRESSO[1,6],1,lower.tail=F))

se2=sqrt(((beta_cor <- resMRPRESSO[2,3])^2)/qchisq(Pval_cor <- resMRPRESSO[2,6],1,lower.tail=F))

resMRPRESSO <- resMRPRESSO %>%

dplyr::mutate(se = c(se1,se2))

#Generate globe test_P and outliers

outliers <- dat$SNP[mr_presso_res[["MR-PRESSO results"]][["Distortion Test"]][["Outliers Indices"]]]

outliers = as.data.frame(outliers)

global_test_p = as.data.frame(global_test_p)

resMRPRESSO

TTT <- plyr::rbind.fill(resMRPRESSO, global_test_p,outliers)

TTT <- as.data.frame(TTT)

TTT

openxlsx::write.xlsx(TTT,file = paste0("Filename/",exp,"-MR-PRESSO.xlsx"), row.names = FALSE)

res=TwoSampleMR::mr(dat,method_list= c("mr_ivw" ,

"mr_weighted_median" ,

"mr_egger_regression",

"mr_weighted_mode"))

print(paste0(exp,"_SNP数_",res$nsnp[1]))

results <- TwoSampleMR::generate_odds_ratios(res)

results$estimate <- paste0(

format(round(results$or, 2), nsmall = 2), " (",

format(round(results$or_lci95, 2), nsmall = 2), "-",

format(round(results$or_uci95, 2), nsmall = 2), ")")

resdata <- dat

openxlsx::write.xlsx(dat,file = paste0("Filename/",exp,"-dat.xlsx"), row.names = FALSE)

names(resdata)

Assumption13 <- subset(resdata,mr_keep==TRUE,

select = c("SNP","pval.exposure",

"pval.outcome", # "F_statistic",

"mr_keep"))

openxlsx::write.xlsx(x = list(

"main"=results,

"Assumption13"=Assumption13),

overwrite = TRUE,

paste0("Filename/",exp,"-res.xlsx"))

}}

if(length(dat[,1])>2){

res_hete <- TwoSampleMR::mr_heterogeneity(dat)

res_plei <- TwoSampleMR::mr_pleiotropy_test(dat)

res_leaveone <- mr_leaveoneout(dat) #

######steiger test######

dat$r.exposure <- get_r_from_bsen(b = dat$beta.exposure,

dat$se.exposure,

dat$samplesize.exposure)

dat$r.outcome <- get_r_from_bsen(b = dat$beta.outcome,

dat$se.outcome,

dat$samplesize.outcome)

res_steiger <- mr_steiger2(

p_exp = dat$pval.exposure,

p_out = dat$pval.outcome,

n_exp = dat$samplesize.exposure,

n_out = dat$samplesize.outcome,

r_exp = dat$r.exposure,

r_out = dat$r.outcome

)

res_steiger <- directionality_test(dat)

p1 <- mr_scatter_plot(res, dat)

p1[[1]]

pdf(paste0("Filename/",exp,"_scatter.pdf"))

print(p1[[1]])

dev.off()

res_single <- mr_singlesnp(dat)

p2 <- mr_forest_plot(res_single)

pdf(paste0("Filename/",exp,"_forest.pdf"))

print(p2[[1]])

dev.off()

p3 <- mr_funnel_plot(res_single)

pdf(paste0("Filename/",exp,"_funnel.pdf"))

print(p3[[1]])

dev.off()

res_loo <- mr_leaveoneout(dat)

pdf(paste0("Filename/",exp,"_leave_one_out.pdf"))

print(mr_leaveoneout_plot(res_loo))

dev.off()

library(magrittr)

res3 <- results[1:3,]

res3 <- res3[,-c(10:14)]

# Convert to paper format

library(magrittr)

# Main result

res4 <- tidyr::pivot_wider(

res3,names_from ="method",names_vary = "slowest",

values_from = c("b","se","pval","estimate") )

# Heterogeneity statistics

res_hete2 <- tidyr::pivot_wider(

res_hete,names_from ="method",names_vary = "slowest",

values_from = c("Q","Q_df","Q_pval") ) %>%

dplyr::select( -id.exposure,-id.outcome,-outcome,-exposure)

# Horizontal pleiotropy

res_plei2 <- dplyr::select(res_plei,

egger_intercept,se,pval)

##steiger

res_steiger2 <- dplyr::select(res_steiger,

correct_causal_direction,steiger_pval)

# Merge

res_ALL <- cbind(res4, res_hete2, res_plei2,res_steiger2)

write.csv(res_ALL,file = paste0("Filename/",exp,".csv"), row.names = FALSE)

}}
